# Prevention of Root Caries Using Oxalic Acid

**DOI:** 10.3390/ma16041454

**Published:** 2023-02-09

**Authors:** Hidetoshi Oguma, Yasuhiro Matsuda, Kumiko Yoshihara, Katsushi Okuyama, Masahiko Sakurai, Takashi Saito, Satoshi Inoue, Yasuhiro Yoshida

**Affiliations:** 1 Division of General Dentistry, Hokkaido University Hospital, Sapporo 060-8586, Japan; 2 Division of Clinical Cariology and Endodontology, Department of Oral Rehabilitation, School of Dentistry, Health Sciences University of Hokkaido, Tobetsu 061-0293, Japan; 3 Health and Medical Research Institute, National Institute of Advanced Industrial Science and Technology (AIST), Takamatsu 761-0395, Japan; 4 Department of Dental Materials Science, Asahi University School of Dentistry, Mizuho 501-0296, Japan; 5 Department of Biomaterials and Bioengineering, Faculty of Dental Medicine, Hokkaido University, Sapporo 060-8586, Japan

**Keywords:** oxalic acid, polyacrylic acid, root caries, hypersensitivity, automatic pH cycle

## Abstract

Certain dentin hypersensitivity treatment materials include oxalic acid to coat dentin surfaces with minerals, while certain organic acids possess a remineralization effect. Herein, an organic acid that inhibits the demineralization and coating of root surfaces was evaluated. Specimens were produced using five non-carious extracted bovines. Four different acids were used: oxalic acid (OA), malonic acid (MA), polyacrylic acid (PA), and succinic acid (SA). Each acid was applied to the root surface and washed using distilled water or a remineralization solution, and the surface was observed using scanning electron microscopy (SEM). All the surfaces of each specimen, barring the polished surface, were covered with wax and immersed in an automatic pH cycling system for two weeks. Dentin demineralization was analyzed using transverse microradiography (TMR) before and after pH cycling. SEM analysis demonstrated that the three acid groups demineralized the dentin surface, whereas the OA group generated crystals covering the dentin surface, even in a distilled water environment. TMR analysis revealed that the OA groups showed significantly lower integrated mineral loss compared with the other groups, even in the distilled water environment. The results suggest that OA generates insoluble calcium oxalate crystals on the dentin and suppresses demineralization even under low saliva conditions.

## 1. Introduction

Untreated dental caries are an acute disease in human healthcare [1,2,3]; yet the percentage of people over the age of 80 with 20 or more permanent teeth is increasing. The maintenance of 20 or more teeth amongst the elderly is associated with healthy aging [4]. Moreover, we expect to witness an increase in the number of remaining healthy teeth in the elderly population in the future. However, preserving these healthy teeth is one of the most critical issues in society presently. In terms of the remaining healthy teeth, gingival recession exposes root surfaces, thereby increasing the risk of root surface caries and dentin hypersensitivity amongst the elderly. Additionally, several older adults suffer from chronic systemic diseases and are prone to xerostomia, which is caused due to the side effects of medications, including antihypertensive drugs.

Moreover, oral cancer and the associated radiation therapy can result in a decrease in salivary secretion, even in the elderly [5,6]. Furthermore, a dry mouth reduces the effectiveness of saliva in cleaning the oral cavity and increases the risk of infections, such as root caries and periodontal disease, by increasing the number of oral bacteria [7,8]. Therefore, the prevention of root surface caries amongst the elderly is a significant issue in oral healthcare. A daily treatment that includes the application of 5000 ppm fluoride, silver diamine fluoride, or high-concentration fluoride is recommended for the prevention of root surface caries in the elderly [9]. For aesthetic reasons, silver diamine fluoride has not been widely used on permanent teeth in the past; however, its efficacy has been re-evaluated in the recent years [10]. As indicated in the guidelines of ADA, it is also used to prevent root surface caries and erosion [9].

Fluoride varnishes, which are used to prevent root surface caries, are employed in dentin hypersensitivity treatments [11]. Fluoride promotes remineralization; however, its substrates, such as calcium and phosphorus, are supplied naturally by the saliva, which aids in remineralization. Therefore, quantitative and qualitative changes in the saliva exacerbate the risk of dental caries and periodontal disease in the elderly and irradiated patients [12]. Therefore, the supply of phosphorus, calcium, and fluoride is essential for the prevention of such dental illnesses. For this reason, fluoride varnishes containing fluoride, casein phosphopeptide-amorphous calcium phosphate (CPP-ACP), and β-tri-calcium phosphate (βTCP) have been applied in the past [13,14]. These materials can simultaneously supply elements such as fluoride, phosphorus, and calcium to the dentin, albeit at a slow rate as the varnish is water-resistant. Additionally, it has even been reported that high concentrations of fluoride, phosphorus, and calcium play a significant role in the remineralization of dentins [15]. 

Meanwhile, certain coating materials containing potassium oxalate are employed to seal dentin tubules with calcium oxalate as a form of treatment for dentin hypersensitivity. Such materials can lead to the rapid formation of a calcium oxalate layer on the root surface using the calcium from the root dentin, which is beneficial for patients with dry mouths. Dicarboxylic acids, such as oxalic and malonic acid, react with calcium to produce calcium compounds [1]. The pH of 0.1 M oxalic acid is 1.26, and its high acidity causes calcium phosphate (a component of teeth) to dissolve easily; however, it instantly reacts with calcium to form calcium oxalate monohydrate. The solubility of calcium oxalate in water is 0.00067 g/100 mL (at 20 °C) [16], which is a minimal value. Potassium oxalate is used as a dentin hypersensitivity suppressant owing to its ability to seal dentin tubules [11,17].

Polyacrylic acid is an organic polymeric acid that displays adhesive properties with glass and calcium. It is commonly used in clinical dentistry as a glass ionomer cement and tooth surface preparation material. In non-classical calcification, polyacrylic acid has also been reported to be present in the cores of mesocrystals [18]. Unlike conventional classical mineralization from a crystal core, non-classical mineralization is a type of biomineralization mineralized from polymers as a core. Tay et al. reported that the non-classical mineralization of dentin induces dense calcification and is also advantageous toward adhesion [19]. Thus, carboxylic acids hold the potential to inhibit demineralization and enhance remineralization.

Acid resistance of dentin hard tissues depends on their composition and structure. Therefore, demineralization and remineralization of teeth also change depending on the pH around the teeth. As the pH of the oral cavity changes continuously, as typified by Stefan’s curve, studying the changes in dentin demineralization and remineralization requires immersion in acids or remineralization solutions and a dynamic simulation of the intraoral pH levels [20,21].

This study aimed to investigate the effects of carboxylic acids on the coating of dentin surfaces and to inhibit demineralization.

## 2. Materials and Method

### 2.1. Specimen Preparation

**Figure 1:**
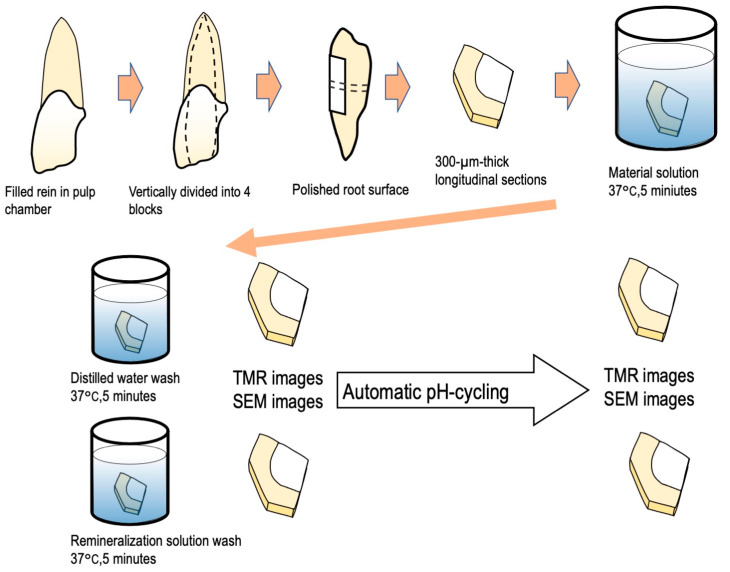
Experimental procedure for SEM and anti-demineralization analysis.

The experimental procedure is outlined in Figure 1.

**Figure 2:**
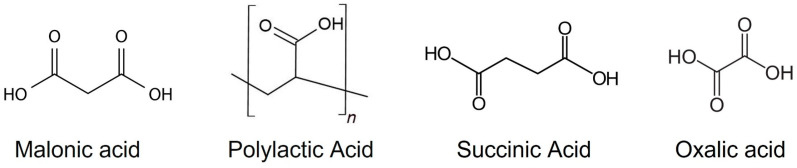
Chemical formulae of the organic acids used in this study.

The fabrication of the bovine deciduous anterior teeth was as follows: epoxy resin (Epofix, Struers, Tokyo, Japan) was poured into bovine-extracted mandibular premolars (n = 5) pulp cavity and stored at 60 °C for 24 h to cure the epoxy resin. Each tooth was longitudinally divided into a four-specimen block using a low-speed rotary cutting machine (IsoMet, Buehler, Lake Bluff, IL, USA). Furthermore, to expose the fresh dentin, they were polished using a manual polisher (EcoMet3, Buehler, Tokyo, Japan) and water-resistant polishing paper (#400–800, Buehler). The specimens were cut perpendicular to the tooth axis into 300 μm-thick sections using a slow rotary cutter. Finally, to prepare single-section specimens, all the surfaces except the exposed dentin surface were coated with a sticky wax. Potassium oxalate monohydrate (Nacalai Tesque, Kyoto, Japan), polyacrylic acid 25,000 (FUJIFILM Wako Pure Chemicals, Osaka, Japan), and malonic acid (FUJIFILM Wako Pure Chemicals, Osaka, Japan), each in 5% solution, were used as the material groups (oxalic acid (OA), polyacrylic acid (PA), succinic acid (SA), and malonic acid (MA) (Figure 2)); deionized distilled water was used as a control (CO).

The bovine tooth single-section samples were immersed in 500 µL of each solution (CO, MA, PA, SA, and OA) for 5 min and then washed three times using 500 µL of deionized distilled water. After the treatment, the groups washed using 500 µL of remineralization solution were designated as (CO_RE, MA_RE, PA_RE, SA_RE, and OA_RE).

### 2.2. Automatic pH Cycling System

pH cycling was conducted following the methods established/used by Matsuda et al., 2006 [20,22]. A Pharmed tube (Saint-Gobain, Tokyo, Japan) with an inner diameter of 3.0 mm was connected to a 10-mL styrene stick bottle (As One, Tokyo, Japan). The microtubing pump (MP-3N, Tokyo Rika Kikai, Tokyo, Japan) connected to the Pharmed tube was controlled (On/Off) using a timer (Labclock, As One, Tokyo, Japan) and it was cycled through the Styrol rod bottle to automatically generate a pH cycle (pH 4.5–6.8) in the vessel. 

The demineralizing solution (pH 4.5) contained 0.2 M lactic acid, 3.0 mM calcium chloride (CaCl_2_), and 1.8 mM potassium dihydrogen phosphate (KH_2_PO_4_); the remineralization solution (pH 6.8) comprised 0.02 M HEPES, 3.0 mM CaCl_2_, and 1.8 mM KH_2_PO_4_ [20]. The contents were cycled through the Styrol rod bottle to automatically generate a pH cycle in the vessel. The average time set for the pH to remain below 5.5 (demineralization time) during each cycle was 18 min. The average time set for the pH to return to the initial pH (recovery time) was 52 min. The number of cycles was six times a day (at 6, 9, 12, 15, 18, and 21:00 h), with an interval of 120 min between each cycle. During this period, when the apparatus was not operating, the specimens were immersed in the remineralization solution. The cycle period was two weeks. Single-section specimens were inserted into a sample pack (Eiken Chemical Co., Ltd., Tokyo, Japan) and placed in a styrene stick bottle for pH cycling.

### 2.3. Scanning Electron Microscopy (SEM) Observation

The treated samples (CO, MA, PA, SA, OA, CO_RE, MA_RE, PA_RE, SA_RE, and OA_RE) (n = 5) were observed on the dentin surface using SEM. Except for the polished surfaces, the samples that did not undergo SEM observation (n = 5) were coated with a sticky wax and then subjected to a 2-week pH cycle, followed by SEM observation. The samples in absolute ethanol were mounted to an aluminum stub with uncoated carbon tape. SEM was used to analyze the particle morphology and size distribution at 30 K and 3 K magnification using an S-4800 (Hitachi) scanning electron microscope at 5 kV. 

### 2.4. Mineral Loss Analysis

The amount of demineralization was evaluated using transverse microradiography (TMR). TMR images were obtained using a soft X-ray system (CSM-2, Softex Corporation, Kanagawa, Japan) with an aluminum step-wedge (AL-013171, Nilaco, Japan 10 µm × 20 steps) and high-resolution photo plates (HRP-SN-2, Konica Minolta, Inc., Tokyo, Japan) before and after two weeks of pH cycling. The exposure was performed for 20 min at 14 kV and 4 mA with a focus-specimen distance of 44 mm. A pulp cavity wall was used to superimpose the TMR images. 

First, digitization was performed using a microscope camera (PCM-500, As One, Tokyo, Japan) connected to an optical microscope (BX50, Olympus, Tokyo, Japan) and a general-purpose image processing software (Adobe Photoshop CS5.1, Adobe Systems, San Jose, CA, USA). Subsequently, digital images before and after the pH cycling were superimposed using the image editing software (Adobe Photoshop Element) with the pulp cavity wall as the reference; furthermore, the area from the superimposed chamber wall with a width of 100 µm toward the depth was extracted as the analysis area. The degree of darkening of the TMR images before and after the pH cycle was analyzed using a general-purpose image analysis software (Image J, Bethesda, MD, USA). The obtained density was analyzed according to the previous report [2] to calculate the mineral profiles of each sample before and after the pH cycle. On the TMR images, the average density of the areas with a width of 50 µm was measured at 0.67 µm increments. Additionally, the integrated mineral loss (ΔIML) was calculated from the mineral profiles obtained before and after the pH cycling according to previously reported procedures [11,23]. From the obtained mineral profiles, the integrated mineral loss (IML) (Vol %×µm) and lesion depth (Ld) were determined were determined, and the difference between the IML (∆IML) and Ld (∆Ld) values before and after pH cycling were calculated. The obtained values were statistically analyzed at a significance level of 5% using one-way ANOVA and Tukey’s HSD test for demineralization (*p* < 0.05).

## 3. Results

**Figure 3:**
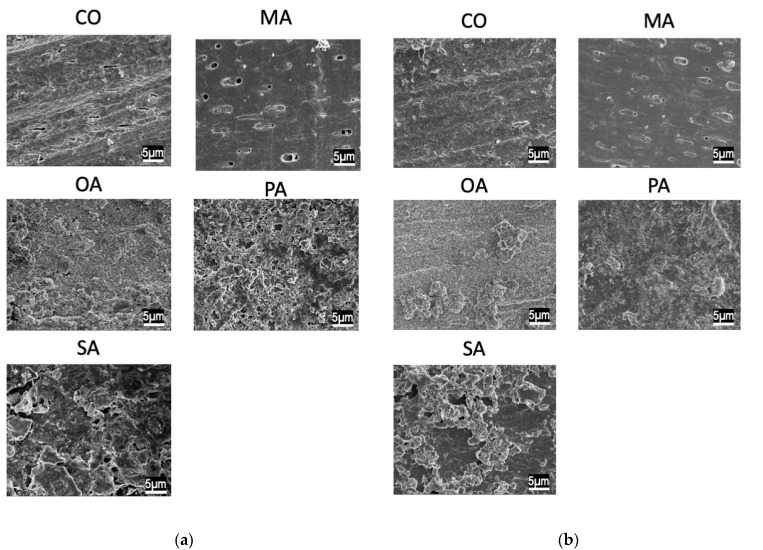
SEM images of each group before pH-cycling. (**a**) Deionized distilled water wash, (**b**) remineralization solution wash (×3000).

**Figure 4:**
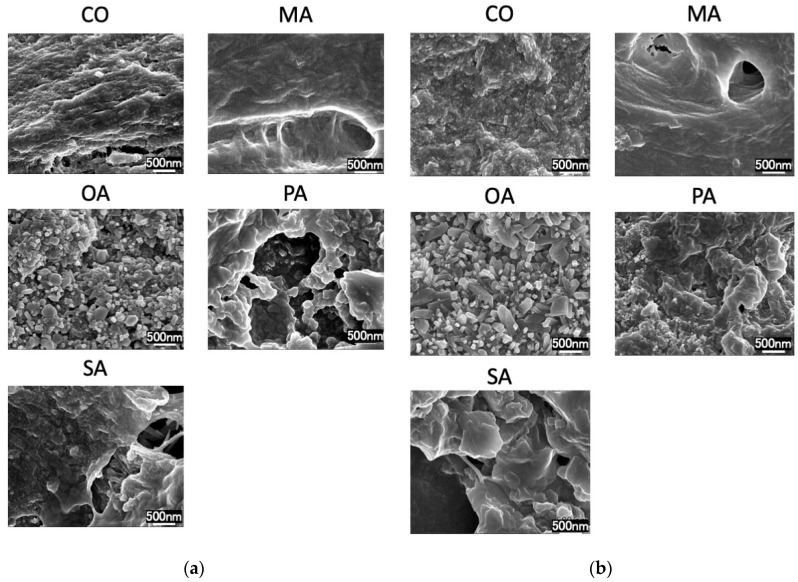
SEM images of each group before pH cycling. (**a**) Deionized distilled water wash, (**b**) remineralization solution wash (×30,000).

**Figure 5:**
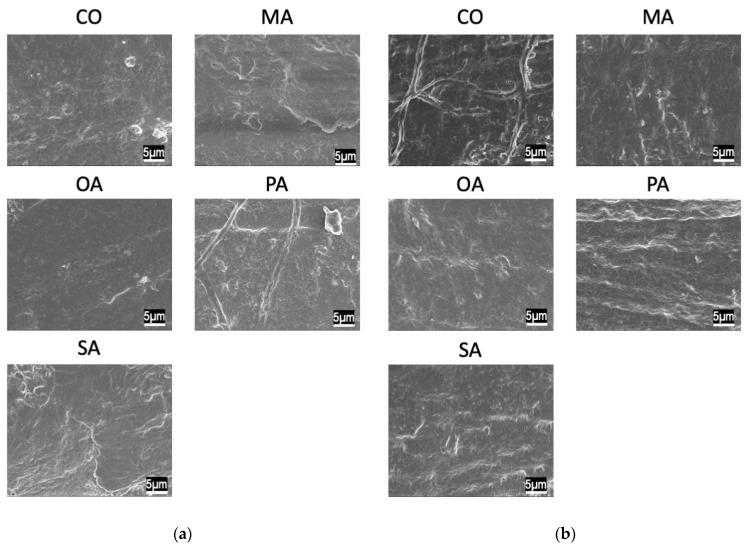
SEM images of each group after pH-cycling. (**a**) Deionized distilled water wash, (**b**) remineralization solution wash (×3000).

**Figure 6:**
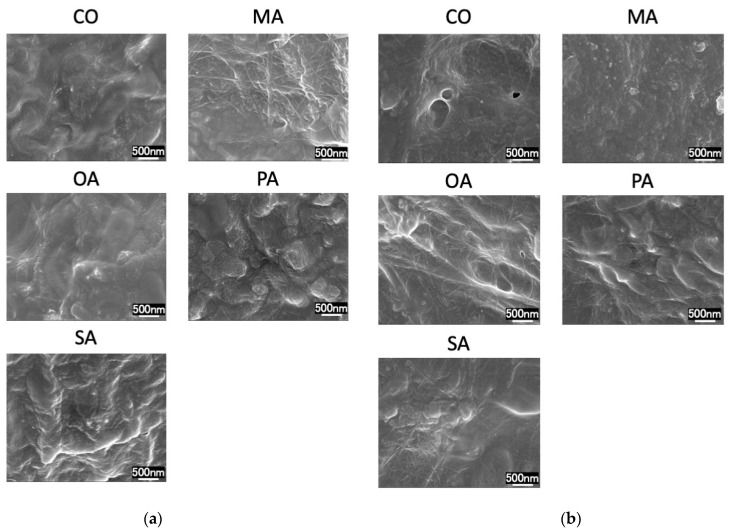
SEM images of each group after pH-cycling. (**a**) Deionized distilled water wash, (**b**) remineralization solution wash (×30,000).

SEM images before the pH cycling are shown in Figure 3 and Figure 4, while those after the pH cycling are shown in Figure 5 and Figure 6.

In the CO group, a smear layer was observed on the surface. Additionally, in the malonic acid group, the opening of the dentin tubules was exposed. In the PA and SA groups, crystals remained around the tubules. Moreover, in the OA group, cubic crystals precipitated densely on the surface layer. The remineralization solution group exhibited the same tendency and did not differ from the distilled water-treated group.

In all the groups, tubules in the dentin surface layer were sealed.

**Figure 7:**
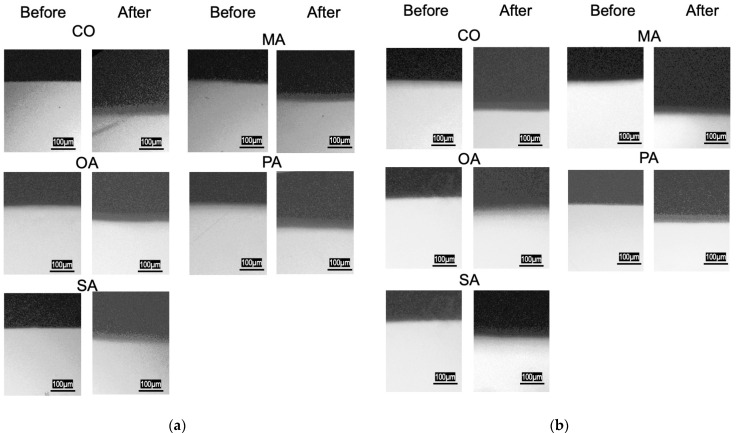
TMR images of each group before and after pH-cycling. (**a**) Deionized distilled water wash, (**b**) remineralization solution wash.

The TMR images before and after pH cycling are shown in Figure 7. 

**Figure 8:**
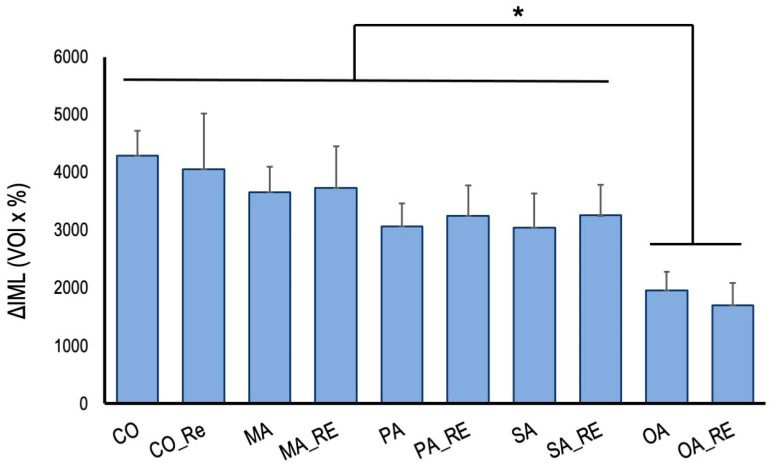
Mineral loss increase differences (ΔIML) after 2 weeks of cariogenic pH cycling. Asterisk significant differences (one-way ANOVA and Tukey’s tests, *p* < 0.05).

The increase in ΔIML after pH cycling is shown in Figure 8. 

**Figure 9:**
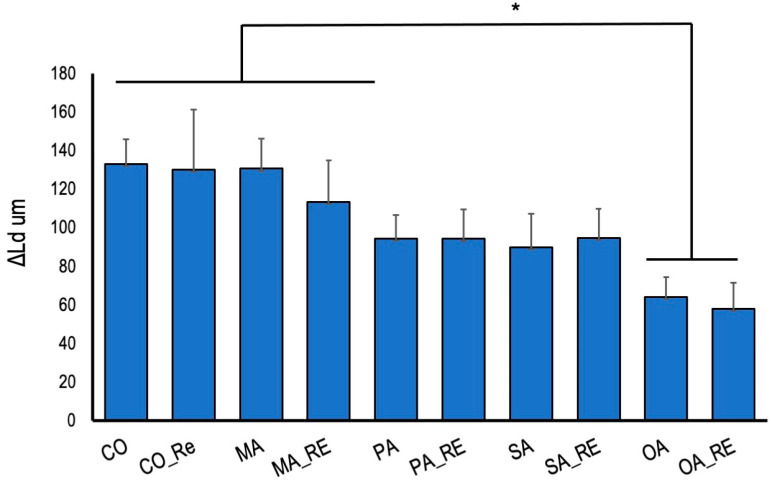
Lesion depth differences (ΔLd) after 2 weeks of cariogenic pH cycling. Asterisk significant differences (one-way ANOVA and Tukey’s tests, *p* < 0.05).

The increase in ΔLd after pH cycling is shown in Figure 9.

The OA and OA_RE groups showed significantly less demineralization than that the control group. The PA and PA_RE, and SU and SU_RE groups exhibited a trend toward lower demineralization. No significant difference was observed between the groups rinsed with the remineralization solution and deionized water; treatment with oxalic acid had a demineralization inhibitory effect regardless of the surrounding environment.

## 4. Discussion

SEM observations revealed a crystalline structure on the surface of the oxalate-treated dentin. Succinic acid and the polyacrylate-treated dentin exhibited residual calcification; however, dentin tubules were observed. It has been reported in various studies that the presence of oxalic acid on dentin surfaces causes calcification of the surface layer and sealing of dentin tubules [24]. This effect is factored in during the preparation of various hypersensitivity control materials [25]. Bonding amongst various dentin hypersensitivity control materials can create an acid-resistant polymer film on the surface layer, while certain high-concentration fluoride materials enhance remineralization. Oxalic acid is characterized by forming a calcium phosphate layer on the root surface. The exposed root surfaces having hypersensitivity symptoms are difficult to dry because: (1) it is difficult to prevent moisture from penetrating the exposed roots and gingiva, and (2) air-drying aggravates the aforementioned symptoms. Additionally, polymeric coatings have dentin-adhesive properties and acid resistance; however, their adhesive properties are reduced due to moisture [26], thereby making the application of polymeric coatings challenging.

High-concentration fluorides, used to treat hypersensitivity, have been widely applied as hypersensitivity control materials because they promote remineralization. According to the Journal of American Dental Association guidelines, high-concentration fluorides are also indicated to be preventive materials for root surface caries [9], and the demineralization-inhibiting effect of such materials has been reported in previous in vitro experiments [11,27]. However, several reports stated that even high concentrations of fluoride do not show considerable demineralization-inhibiting effects in weakly acidic artificial saliva [7]. Moreover, the environmental factors are expected to play a significant role in the demineralization-inhibiting and remineralization effects of dentin [12].

In this experiment, dentin, treated with oxalic acid on the dentin surface, was washed with a remineralization solution; however, the surface structures were not different from those when washed with distilled water, suggesting that the surface layer was covered with calcium structures from the dentin. Previous studies have shown that the surface structures contain calcium oxalate [28], crystallizing at pH levels between 4.5 and 5.5 [29], which is lower than the critical pH of dentin and closer to that of enamel. Thus, it can be concluded that surface treatment with oxalic acid rapidly produces a highly acid-resistant layer on the dentin surface. Certain studies have reported on the oxalic acid layer being thin; thus, it has low abrasion resistance and is instantly removed [30]. However, this is unlikely to occur in areas where brushing is difficult and plaque is likely to stick to the surface. The crystal structure is considered to be that of calcium oxalate. The insoluble calcium oxalate on the surface layer of the dentin may have inhibited the demineralization of dentin during the pH cycle. In patients with xerostomia, saliva secretion is low; therefore, the effect of washing away the oral bacteria is less, and remineralization by saliva is not expected. In this respect, oxalic acid is advantageous in terms of creating a highly acid-resistant layer based on the minerals in the dentin without the presence of saliva.

The pH cycle simulates the pH changes in the oral cavity and provides demineralization stress on the dentin surface. Therefore, in the balance between the short demineralization and long remineralization times, the highly acid-resistant calcium oxalate layer might have inhibited the progression of demineralization in the early phase of the pH cycle and reduced the amount of mineral leaching. The SEM images after two weeks of pH cycling showed that the surface dentin tubules were in all groups, suggesting that the surface layer was covered due to calcification caused by the remineralization after demineralization.

In the teeth and hard bone tissues, existing small biomolecules (e.g., citrate and succinate) and biopolymers (e.g., chondroitin sulfate) have been observed to bind to collagen fibers, which improve collagen biomineralization or collagen mineralization [31,32]. In dentin, succinic acid has been reported to promote the remineralization of dentin [33]. SA has been studied as a bioactive biomolecule that can be used for calcium mineral growth control and hard tissue repair. In addition, it has been reported to chemisorb on crystal surfaces through calcium atom coordination to control the calcium mineral growth and morphology [17]. During the mineralization of collagen fibers, SA modification promotes the infiltration or wetting of the calcium ions or amorphous calcium phosphate precursors, thereby creating a high local supersaturation state with high concentrations of calcium and phosphate around the collagen fibers, promoting hydroxyapatite (HAp) nucleation and growth. Thus, SA may promote HAp crystal formation within the collagen fibers and dentin and its intrafibrillar mineralization effect.

Polyacrylic acid nucleates and promotes the formation of mesocrystals in non-classical calcification; furthermore, the presence of PA induces crystals with normalized crystallinity [18]. Biomimetic mineralization has been developed through the binding of PA to collagen fibrils. The PA-doped collagen can guide the scale and distribution of apatites [34]. A low-molecular weight PA is used to create metastable amorphous calcium phosphate (ACP) nanoprecursors [35]. In dentin calcification, remineralization of phosphate-etched human teeth using a Portland cement/phosphate-containing solution system was validated, and the results showed that a phosphate-containing solution with PA produced stable amorphous calcium phosphate nanoprecursors; additionally, remineralization with the mineral trioxide aggregate (MTA) cement was more effective than that with PA. It has been reported that MTA cement remineralization is accelerated by PA [34]. Thus, SA and PA have been reported to promote collagen-centered biomineralization, which in turn promotes dentin remineralization. In the present study, no evident remineralization image was observed in the group rinsed using the remineralization solution; however, compared with MA, an image showing the suppressed demineralization was observed, suggesting that a longer remineralization environment may promote biomineralization.

Compared with the distilled water rinsing group, the demineralization suppression effect tended to be stronger when oxalic acid was rinsed using the remineralization solution. It is considered that washing with the remineralization solution might have caused further calcification, thereby forming a thicker calcium oxalate layer, which might have enhanced the demineralization-inhibitory effect. When the dentin was washed with the remineralization solution, the solution was observed to turn cloudy due to the reaction with the residual oxalic acid. The results showed an inhibitory effect on dentin demineralization and suggested the possibility of a preventive effect on the root surface caries. Chemically, dentin is 65–70% inorganic and 18% organic, shows low acid resistance, and its demineralization proceeds approximately twice as fast as that of enamel [4]. Additionally, fluoride and other ions can easily penetrate deep into the dentin because of the movement of the aqueous solution through the dentin tubules [36]. Thus, the characteristics of the dentin differ from that of the calcium phosphate.

As calcium oxalate calcifies even at low pH levels, the calcium oxalate formed on the surface of dentins can be expected to inhibit demineralization owing to its acid resistance and adhesive properties with calcium in clinical practices. 

## 5. Conclusions

SEM observations and TMR analyses of oxalic acid, malonic acid, and polyacrylic acid on the dentin suggest that oxalic acid generates insoluble calcium oxalate crystals on the dentin, thereby suppressing demineralization even under low saliva conditions.

## Data Availability

Not applicable.
